# Potential point of care tests (POCTs) for maternal health in Peru, perspectives of pregnant women and their partners

**DOI:** 10.1186/1742-4755-11-5

**Published:** 2014-01-16

**Authors:** Angela M Bayer, Lizzete Najarro, Mercedes Zevallos, Patricia J García

**Affiliations:** 1 David Geffen School of Medicine, University of California, Los Angeles, Los Angeles, USA; 2 Facultad de Salud Pública y Administración, Universidad Peruana Cayetano Heredia, Lima, Peru

**Keywords:** Point-of-care diagnostic tests, Maternal health, Antenatal care, Community members, Peru, Qualitative

## Abstract

**Background:**

Globally, no qualitative studies have explored the perspectives of women and their partners about the integration of technology – and specifically diagnostic testing technologies – into antenatal care. The study objective was to describe the demand side for pregnancy-related diagnostic tests from the perspective of Peruvian consumers, including female and male community members, by engaging participants about their awareness of and care-seeking for pregnancy-related diagnostic tests and their preferred characteristics and testing conditions for pregnancy-related point-of-care diagnostic tests (POCTs).

**Methods:**

Sixty-seven mothers and fathers of children under one from the peri-urban coast and the peri-urban and rural highlands and jungle of Peru participated in ten focus groups.

**Results:**

Participants think that pregnancy-related diagnostic tests are important and they and their fellow community members are committed to ensuring that pregnant women receive the tests they need. Participants expressed clear demands for pregnancy-related POCTs, including important characteristics for the tests themselves (certification, rapid, reliable results) and for test implementation (well-trained, personable good communicators as test administrators at well-equipped, convenient testing sites). Participants emphasized the importance of short waiting times and explained that many people have some ability to pay for POCTs, particularly if they are innovative, rapid or multiplex.

**Conclusions:**

Engaging future POCT users as consumers who are able to make key decisions about the development and implementation of pregnancy-related POCTs is valuable and informative.

## Background

Antenatal care (ANC) is key to improving maternal and newborn health around the globe [1]. ANC represents an opportunity to link pregnant women to health services, which are especially important for institutional delivery and postpartum care for women and their newborns [1]. In 2005–2011, 95% and 76% of pregnant women from upper and lower middle-income countries, respectively, reported receiving at least one ANC visit and 53% of pregnant women in lower middle-income countries reported four or more ANC visits [2].

ANC should be offered in a standardized manner and provide a package of interventions that includes screening and treatment for key health conditions. Screening and treatment for certain conditions have been proven effective, e.g. for anemia, syphilis and HIV. The World Health Organization (WHO) currently recommends anemia and syphilis screening as part of essential ANC and HIV testing as situational ANC, based on the community’s disease patterns [1,3]. In Peru, ANC is offered nationwide. National guidelines recommend two ANC schemes: optimal care, with initiation of ANC as early as possible during pregnancy, with monthly visits up to 32 weeks of gestation, biweekly visits in weeks 32–36 and weekly visits from week 37 forward; and minimum necessary care, with two visits prior to 22 weeks, visit 3 at 22–24 weeks, visit 4 at 27–29 weeks, visit 5 at 33–35 weeks, and visit 6 at 37–40 weeks [4]. In 2012, 60% of ANC in Peru was provided by *obstetras*[5]*,* university-educated midwives who are trained to provide comprehensive sexual and reproductive health services with a strong focus on health promotion and prevention. During ANC, health promotion and prevention – according to national guidelines – includes educating pregnant women about how to identify alarm signs and actions to take upon seeing signs; physically and mentally preparing the pregnant woman and her family for birth; creating an individualized birth plan for the pregnant woman that includes participation by the partner, family and community; and promoting family and social support for ANC [4].

National guidelines require the following package of diagnostic tests during the first ANC visit: anemia testing, syphilis testing, HIV testing, blood type and Rh factor testing, diabetes testing, and tests for urinary tract infections and proteins in the urine [4]. Use of HIV point-of-care tests (POCTs) in ANC in certain areas of Peru began in 2004 through initiatives supported by the Global Fund to Fight AIDS, Tuberculosis and Malaria. HIV POCT use in ANC expanded nationwide in early 2005 following a Ministerial Resolution that specified that HIV POCTs could be used in ANC and applied by the health personnel providing ANC [6]. However, for several years, most HIV POCTs were incorrectly implemented using venous blood at central laboratories [7]. The introduction of syphilis POCTs in ANC in Lima started in 2010 with SWAN (CISNE in Spanish, for Immediate Cure for Neonatal Syphilis), the Peru arm of a multi-country operations research study to implement syphilis POCTs [7]. Following successful implementation of SWAN, study personnel worked with policymakers to integrate syphilis POCTs into the updated Peruvian “Guidelines for the Prevention of Mother-to-Child Transmission of HIV and Syphilis” and syphilis POCTs are now offered nationwide [7].

Several qualitative studies with women and health providers have engaged these groups about facilitators and barriers to accessing ANC. Most of these studies were included in a recent meta-synthesis [8] that focused on the barriers side by exploring why women in different contexts do not access ANC. Another key qualitative study engaged women from low- and middle-income countries that participated in a large WHO trial to evaluate a new ANC model. The trial showed that a reduced number of ANC visits using this new ANC protocol led to similar maternal and perinatal outcomes as more ANC visits [9]. The new protocol tested in the trial is now the ANC model recommended by the WHO [3]. The qualitative study that accompanied the trial explored women’s perceptions regarding pregnancy and focused on their experiences while attending (versus not attending, as in the meta-analysis above) ANC visits [10].

Qualitative studies that explore the perspectives of women and their partners about the integration of technology – and specifically diagnostic testing technologies – into ANC are important. It is also important for these studies to approach women and their partners as “consumers” – who provide opinions about product preferences and evaluate available products before selecting the product they will use - and not as ANC “clients” or “patients” – who accept what is available. Therefore, the objective of this study was to describe the demand side for pregnancy-related diagnostic tests from the perspective of Peruvian consumers, including female and male community members. We engaged participants about their awareness of and care-seeking for diagnostic tests during pregnancy and their preferred characteristics and testing conditions for pregnancy-related POCTs.

These data collection activities were carried out as part of a larger multi-disciplinary research project, Brighter Futures, which uses different methodologies to evaluate the needs, costs, barriers and opportunities that influence the introduction of POCTs for pregnant women in Peru and to develop an innovative model for the implementation of POCTs for maternal health in the country. Brighter Futures strives for “integrated innovation,” as described by Singer and Brook, through “the coordinated application of scientific/technological, social and business innovation to develop solutions to complex challenges” [11].

## Methods

### Study setting

Peru, located in South America, has a total population of 29 million. Since Peru has great geographic and cultural diversity, Brighter Futures works in three sites, which represent the country’s three primary geographic zones. Ventanilla, located on the coast and part of metropolitan Lima, is home to 75,000 people and is 100% urban. Quispicanchis, in the Andean highlands in the Cusco region, has 30,000 people, 84% of whom live in rural areas. Yurimaguas, in the jungle in the Loreto region, has a population of 63,000 that is primarily urban (77%).

In Peru, there are several health systems. The public sector Ministry of Health or MINSA is the largest system and covers 76% of the Peruvian population. The semi-public social security health system, EsSalud, is a joint employer-employee funded health system that covers 20% of Peruvians. The Armed Forces and National Police health systems and the private sector cover the remaining 4% of the population [12].

### Study participants

Males and females from Ventanilla, Quispicanchis and Yurimaguas participated in this study. Participants were selected purposively. First, the study team worked with local collaborators (formal and informal community leaders and health providers) to identify parents of children under one year of age. Next, the study team member went with the local collaborator to invite potential participants to the focus group. The study focused on parents of children under one since they could provide in-depth information about the topics of interest.

### Data collection activities

We held ten focus groups, seven with women and three with men. We held separate groups with women and men for two reasons: women are direct users and men are indirect users of maternal health POCTs; and so that women and men would feel comfortable talking about the different topics. The semi-structured field guide addressed the following themes: pregnancy and delivery experiences in the community, including care seeking; awareness of, decision making around, experiences with and perceptions of diagnostic tests during pregnancy; and perspectives on POCTs for maternal health. Regarding perspectives on POCTs, we began by presenting what POCTs are and by showing two examples, of a rapid finger prick-based HIV POCT and a rapid oral saliva-based HIV POCT. We explained that these are only two examples of POCTs for one condition and that POCTs can test for different conditions, have different designs and formats, and take differing amounts of time to produce results. We also mentioned that some POCTs can test for several conditions at the same time. Then, we asked participants which pregnancy-related tests they know about (unprompted awareness of tests demonstrates some level of importance) and which tests they think are most important. We also inquired about the following for POCTs for maternal health: 1) the characteristics that POCTs should have; 2) the types of samples participants know about, propose and would prefer; 3) how quickly they think results should be available; 4) the appropriate people to administer POCTs; 5) places where POCTs could be administered; 6) and whether and how much participants and people they know would be willing to pay for single- and multiple-condition POCTs.

All activities took place at a location that was comfortable for participants. Focus groups were facilitated by a Peruvian professional with extensive experience in qualitative data collection with diverse populations throughout Peru who is committed to engaging populations about their perspectives and experiences in a non-leading manner, without prompting participants to respond in a certain way. The focus groups were audio-recorded and later transcribed verbatim.

### Data analysis

All qualitative data were explored using thematic analysis. We read all of the transcripts and developed an initial set of codes. Next, four team members coded two transcripts to agree on the coding approach and finalize the codebook. Then, two team members coded each of the transcripts using ATLAS.ti 6 (Scientific Software Development GmBH, Berlin). Finally, we synthesized the data by looking for similarities and differences across focus groups.

### Ethics issues

The study protocol and instruments were approved by the IRB of the Universidad Peruana Cayetano Heredia. All participants provided verbal informed consent to both participate in and audio record the focus group prior to initiating their participation.

## Results

Sixty-seven individuals from peri-urban Ventanilla and from peri-urban and rural Quispicanchis and Yurimaguas participated in this study. Participants included 50 women and 17 men who were parents of children under one, as shown in Table 1.

**Table 1:** Focus group participants, by study site

	**Focus groups with women**		**Focus groups with men**	
**Geographic zone/study site**	**Number of groups**	**Number of participants**	**Number of groups**	**Number of participants**
***Coast/Ventanilla***				
Peri-urban	2	16	1	5
Educational level				
Some primary or less		3		2
Some secondary		7		1
Complete secondary		6		2
***Highlands/Quispicanchis***				
Peri-urban	1	7	1	6
Rural	1	6	0	0
Educational level				
Some primary or less		5		4
Some secondary		4		1
Complete secondary		4		1
***Jungle/Yurimaguas***				
Peri-urban	2	18	1	6
Rural	1	3	0	0
Educational level				
Some primary or less		7		1
Some secondary		11		1
Complete secondary		3		4
**Total**		**50**		**17**

The results from the focus groups follow, organized by theme and sub-theme and supported by participant quotes. Participant quotes end with information to show whether the focus group was with females or males, the name of the site and the geographic zone. Table 2 also shows a summary of the link between 1) the specific study objectives, 2) the topics explored in the focus groups, 3) the themes and 4) sub-themes.

**Table 2:** Specific objectives, topics explored and resulting overall themes and sub-themes

**Specific objective**	**Topics explored during focus groups**	**Resulting overall theme**	**Resulting sub-themes**
1. To explore how participants perceive and value pregnancy and ANC, since this overall perception influences how individuals perceive pregnancy-related POCTs	• Pregnancy experiences in the community	Good pregnancy-related health and quality ANC, including diagnostic tests, are key to improved maternal and child heath	• Pregnancy is an important, shared experience
	• Awareness of, decision making around, experiences with and perceptions of pregnancy-related diagnostic tests		• Pregnancy-related diagnostic tests are valuable for mothers and children
			• Male partners and health providers are vital to ensuring pregnant women receive diagnostic tests
2. To explore how participants perceive pregnancy-related POCTs and their preferences for pregnancy-related POCTs	• Characteristics that POCTs should have	Participants have clear preferences regarding pregnancy-related POCTs	• Participants prefer tests for anemia, urinary infections and HIV
			• Participants want tests with "certification," blood-based samples and rapid and reliable results
	• Types of samples participants know about, propose and prefer		
			
			• Well-trained, personable good communicators should administer tests
	• How quickly results should be available		
	• Appropriate people to administer POCTs		• Tests could be performed at different places
	• Places where POCTs could be administered		• Short waiting times at the testing site are critical
	• Whether and how much participants and people they know would be willing to pay for single- and multiple-condition POCTs		• Many people have some ability to pay, particularly for multiplex tests

### Good pregnancy-related health and quality ANC, including diagnostic tests, are key to improved maternal and child heath

Female and male participants from urban, peri-urban and rural areas of the three study sites clearly understand that good health during pregnancy and quality ANC – and particularly diagnostic tests – are key to improving the health of mothers and children in their communities. While all participants recognized the importance of pregnancy-related diagnostic tests, participants from the highlands and jungle emphasized that health providers and partners play an important role in ensuring that pregnant women get tested.

### Pregnancy is an important, shared experience

All participants described significant collaboration during pregnancy given that it is an important period in the lives of all families. Participants affirmed that women tend to take care of themselves, with support from their mothers or older children. They also receive a lot of support from their male partners. If men do not accompany their pregnant partners during their ANC visits, it is due to work responsibilities, not due to lack of interest.

*Facilitator: When women are pregnant, who helps them?*

Participant 1: Usually you spend time with your partner… Sometimes we don’t live with our mothers.

*Facilitator: Do your partners go with you for your antenatal visits?*

Several participants together: Not usually… but because of work.

Participant 2: In their free time, they go with us.

*Facilitator: Are there fathers who don’t go?*

Participant 2: No… none of that.

Participant 1: It’s because of their work.

*Participant 3: When they have free time, they* ***do*** *go.*

(Focus group with women, Yurimaguas, jungle)

### Pregnancy-related diagnostic tests are valuable for mothers and children

Focus group participants from all the groups reported that pregnant women undergo diagnostic tests for two reasons: for the good of their babies and their families and to detect and treat problems in a timely manner. It is important to underscore that several women mentioned that they would not get tested if they were not pregnant. Additionally, in the highlands, participants mentioned that women are more likely to get the recommended tests if it is their first child.

Participant 1: When I’m pregnant, I do get all of the exams, but if I’m not, [I get] nothing.

Participant 2: Of course, since they [health providers] ask me to get them [tests], I have to do it for my baby.

(Focus group with women, Ventanilla, coast)

When we go to our check-ups, well, when I went to my check-ups, the woman asked us, “you’re going to get these analyses to see if you’re okay. Because if you’re sick… you can transmit it to your baby.” And since it’s my first child, I have to do the analyses.

(Focus group with women, Quispicanchis, highlands)

### Male partners and health providers are vital to ensuring pregnant women receive diagnostic tests

In some cases and in particular in the jungle and highlands, male partners have to demand – in a positive sense, for the good of the woman and the baby – that women get the recommended tests.

Participant 1: My husband always sent me, “Go, get your check-up. If you’re okay, the baby is okay”.

Participant 2: Yes, it was the same for me.

(Focus group with women, Quispicanchis, highlands)

Participant 1: They [women] always get nervous. Sometimes they say no… but that’s why we’re here, to be able to say to them, “yes, you have to do it [get tested]”.

Participant 2: If the father isn’t around, she has to do it anyway. In my case if I’m not here, of course she has to get them [the tests]… not wait to ask.

(Focus group with men, Yurimaguas, jungle)

Female focus group participants described how health providers in the jungle and highlands have to intervene in some cases, by going to pregnant women’s homes to encourage the women to come to the health establishment to have the tests done.

### Participants have clear preferences regarding pregnancy-related POCTs

Prior to engaging participants about their preferences regarding pregnancy-related POCTs, the facilitator explained what POCTs are and provided a few examples of existing non-maternal health POCTs, so as not to unduly influence participants. Then, the facilitator asked participants about their preferences for maternal health POCTs. When first asked to discuss their preferences, participants were hesitant since they are used to being treated as “patients” who accept what is available. Once the facilitator clarified that participants could state any and all preferences, they expressed several.

### Participants prefer tests for anemia, urinary infections and HIV

Focus group participants named three pregnancy-related diagnostic tests as most important: anemia, urinary infection and HIV. Their reasons for preferring these three tests were that they were the most common tests that participants or participants’ partners had undergone during their current or previous pregnancies. Additionally, female participants reported that urinary infections during pregnancy were very frequent.

### Participants want tests with “certification,” blood-based samples and rapid and reliable results

#### ***Test “certification”***

Focus group participants were asked to propose and discuss the characteristics that they felt are most important to have in POCTs for pregnant women in Peru. Participants underscored the importance of product “certification,” understood as both 1) a seal or stamp on the test package and 2) a seal on the test result, to demonstrate that the test has been certified by the appropriate international organization.

I think that it [a test] is like any type of device that is sold… it always has to have a certification, [which shows] that it is suitable to provide a good result. I think that if it has this certification, the result will be correct… The tests… should have a printed international certification, to show that they are reliable.

(Focus group with men, Yurimaguas, jungle)

#### ***Preferred type of sample for tests***

Focus group participants brainstormed several types of samples, including blood, saliva, urine, hair, breath, foot and fingernail- or toenail-scrapings. However, when asked which type of sample they would trust most, everyone agreed on blood. In terms of the type of blood sample, most participants preferred a finger prick over venous blood. Some considered venous blood to be advantageous since it is possible to get a larger quantity of blood and be able to carry out several analyses. However, it is also painful. They considered finger pricks to be faster and less painful, but stated that they fear that sometimes they do not produce enough blood and that only one analysis is possible. When discussing types of samples, participants emphasized the importance of good explanations regarding the test and its utility during the testing process. The test administrator should explain, for example, why a finger-prick blood sample can provide a sample that is as useful as a venous blood sample.

#### ***Rapid and reliable results***

One common theme in all of the focus groups was the peoples’ request and preference for tests that provide fast results and are also reliable. In terms of rapid test results, participants stated that “rapid” means 15–30 minutes. Participants said that some results may take more time and that if they do, the test administrator should explain why and should specify the delay; regardless, the result should be ready within the same day.

*Facilitator: How long would you be able or would you like to wait to get your [test] results?*

Participant 1: It could be 30 minutes.

Participant 2: It could be. We don’t know. For example, maybe they need more time. It would be better for them to explain it to us, right? To know that they’re going to take the sample quickly but that [the result] will be ready in this [specific] amount of time. They should explain it.

(Focus group with women, Quispicanchis, highlands)

Participants also mentioned that if a result is ready too quickly (in minutes), a person might question its validity. Therefore, it is important to explain that a rapid test can be as good as a non-rapid test.

### Well-trained, personable good communicators should administer tests

Participants proposed and described who they thought would be the most appropriate people to perform diagnostic tests. Participants named training, experience, good rapport with clients, and the ability to explain the testing process and results as abilities that are more important than the person’s sex or profession.

Participant: A health professional, a doctor, a technician… The important thing is for the person to have knowledge about the issue.

*Facilitator: And could it be a [health] promoter?*

Participant: If the person has training, why not?

(Focus group with men, Ventanilla, coast)

Focus group participants emphasized the importance of brief yet thorough explanations both prior to taking the sample and when delivering the result. Prior to sample taking, the test administrator should explain how the sample will be taken, brief detail about how it will be processed, when the result will be ready, and how the person can obtain the result. When results are returned, the test administrator should clearly explain the result, whether follow-up or treatment is necessary, and other steps that the person should take in the future.

In the highlands, participants also felt that the provider’s ability to speak the local language Quechua is key so that they can receive their test results in their own language.

Sometimes they [providers] don’t understand. Sometimes people that don’t know Quechua, they get frustrated, they say, “what are you saying to me?” The nurses can get angry… so the husband who speaks both languages, Quechua and Spanish, explains everything… Some [clients] don’t know Spanish, it would be hard [for them] to get care.

(Focus group with men, Quispicanchis, highlands)

### Tests could be performed at different places

In terms of where diagnostic tests could be implemented, participants mentioned public and private health establishments, pharmacies and boticas, community centers and schools, and even their own homes. Additionally, the place should be clean, spacious and fully enclosed (with well-sealed walls and a roof) and have all of the necessary equipment and supplies. In Yurimaguas, participants also mentioned the importance of running water and electricity.

In the case of communities, it could be at the community center with the health promoter… if… the promoter has received some type of training.

(Focus group with women, Quiquijana, highlands)

An adequate environment, which has electricity, water, seats… very safe, well-enclosed like a mini-laboratory… a place that is not too exposed to heat…

(Focus group with men, Yurimaguas, jungle)

Participants also emphasized the importance of “access”: the testing site should be close to (or at) their homes. Particularly in the highlands and jungle, participants emphasized that tests should be administered in their communities since they live far away from health establishments. As a male participant from the highlands said, *They [the Ministry of Health] even train people [that live at high] altitudes… The doctor trains the [health] promoter…*

Participants preferred diagnostic testing at their own homes above all other options.

At home… Getting to the health post is too far… Doctors also can’t get there quickly [and] they don’t see you quickly.

(Focus group with men, Yurimaguas, jungle)

### Short waiting times at the testing site are critical

Most female participants sought ANC in the public sector, the Ministry of Health or MINSA. Some participants in Ventanilla also used the semi-public social security health system, EsSalud. In Ventanilla, women were able to access EsSalud if their male partners had formal-sector jobs.

In both MINSA and EsSalud, participants reported significant waiting times. In MINSA, if a woman is willing to arrive early (5–6 am) and spend 2–6 hours at the establishment, she will be able to get same- or next-day care. There are waits at each point in the care process: to obtain a ticket for care, to pay the cashier, to request her clinical history and to wait for her appointment (the longest delay). If the woman is unable to obtain a ticket for care, she has to return the next day and start the process again. In EsSalud, women also need to wait to make an appointment. However, the appointment would be days, weeks or even months later.

Participants presented the private sector, which has higher out-of-pocket payments than MINSA or EsSalud, as a model for short waiting times. There, the entire care process is much simpler and faster. There is no need to make an appointment or obtain a ticket for care. The person goes directly to pay the cashier and then waits a short time (5–10 minutes) for the service, in this case, the diagnostic test.

In the private [sector], it’s fast… [you] pay, [in] one minute…Whenever I’ve gone, it’s fast, no lines… At that same time, I go to the laboratory, the same day… 15 minutes [total].

(Focus group with women, Ventanilla, coast)

### Many people have some ability to pay, particularly for multiplex tests

Participants mentioned that cost is usually not a consideration in pregnancy care since most services are free of charge since: 1) women qualify for the Comprehensive Health Insurance (*Seguro Integral de Salud* or SIS), which subsidizes MINSA services for poor and extremely poor pregnant women; or 2) women are covered by EsSalud.

Despite the availability of free services, participants in all focus groups named the private sector as an important source of care for pregnancy-related diagnostic tests. Participants reported that many people in their communities have some ability to pay for diagnostic tests. They said that they would be willing to pay particularly for innovative tests such as rapid tests and that they would be willing to pay 2–3 times more for multiplex tests.

Additionally, 1–2 participants in each of the female focus groups (about 20% of female participants) had gone to the private sector and paid out of pocket for at least one diagnostic test during their last pregnancy. The most common test that women sought out in the private sector was the ultrasound, which can be quite costly. In the three sites, participants reported having paid 15–45 soles (2012 US$ 6–17) for their last ultrasound. A few women in Ventanilla had paid up to 200 soles (over 2012 US$ 75) for a 4D ultrasound.

When tests involve costs, pregnant women and their partners decide together which tests to seek out and where to go.

[The woman] always asks her husband… The doctor… gives her the order and she goes home, asks her partner and they reach an agreement… She has to come and ask the husband because they don’t do all of the analyses at the hospital and you have to go to get them at a private place (establishment).

(Focus group with men, Yurimaguas, jungle)

## Discussion

Participants in our three sites think that pregnancy-related diagnostic tests are important and are committed to ensuring that pregnant women receive the tests they need. Participants expressed clear suggestions for pregnancy-related POCTs, including characteristics for the tests themselves and for test implementation. If the pregnancy-related POCTs meet their demands, participants expressed that they would use these tests and may even be willing to pay.

This study has limitations. First, the data collection method was focus groups. Since focus groups take place in a group setting, the perspectives that emerge tend to represent group norms. However, since our objective was to describe what consumers think about and want in maternal health POCTs, we wanted to gather such norms and explained to participants that we wanted to hear about both their perspectives and those of other people in their community in order to do so. Second, the discussion of POCTs may have been hypothetical or abstract at times since we asked participants to discuss products and implementation scenarios that do not yet exist either globally or in their communities. Prior to and at all points during the focus groups, however, we explained that the focus groups were exploratory and that there were no correct responses. We also decided not to present more than two examples of POCTs so as not to unduly influence participant perspectives since we wanted participants to be creative and unrestricted in their responses.

Despite these limitations, we have several important findings. Participants here demonstrated that they are saavy consumers of POCTs for maternal health and that their preferences match current demands in POCTs for ANC. The conditions they named as most important to screen for in POCTs are anemia, HIV and urinary infections. This runs parallel to recent expert recommendations for POCTs for ANC in developing countries, ideally through a multiplex POCT [13]. Participants also mentioned key characteristics for the tests themselves, including certification that demonstrates that the tests are high-quality. Determination of the quality of tests should be undertaken according to some type of regulatory standards. These issues were also mentioned in the expert review cited above, specifically, the importance of harmonized regulatory standards for POCTs. If harmonization existed, companies could secure agreements to register their POCT products in multiple countries at the international or regional levels instead of having to conduct trials in every country. Harmonization would reduce costs for countries, companies and consumers and would also diminish substandard practices with POCTs in developing countries. For example, developing country markets are currently inundated with the sale, purchase and use of low-quality, unregistered POCTs since companies with high-quality POCTs, and the accompanying commitment to follow individual country’s product registration guidelines, cannot compete [13].

Participants also shared their perspectives on the testing process, where the key factor was good information that addresses certain complexities of tests and the testing process. Participants specified that they want explanations at each step in the testing process: information about what will happen prior to taking the sample, how long results will take, what the results are and what they mean, and next steps including follow-up and treatment. Focus group participants mentioned that they want complexities to be explained when it makes sense to do so; in other words, they want to understand why the test that the institution is using is the most appropriate one for them and others like them instead of feeling that they’re receiving a test because it is the only one available. For example, they would prefer rapid test results to be ready in 15–30 minutes. They understand, however, that some results take longer. Therefore, the test administrator should clearly explain that a test result ready in 20 minutes, for example, can still be as valid as the previous test that took hours or days, as long as the test is of high quality. If the result still takes hours versus minutes, the test administrator should also explain why that amount of time is necessary. These recommendations could be addressed through two mechanisms: adequate training of POCT implementers and client education. The expert review on POCTs also noted the importance of educating health providers on how to use and interpret POCTs and of updating providers on the state of the art in the diagnostics field [13]. For clients, the use of educational materials such as flipcharts with many illustrations and little text could help to communicate these more complex messages in a population-friendly manner and enhance consumer understanding and buy-in. This information “requirement” could also be combined with the harmonization of standards recommendations. For example, if Peru subscribes to regional or international POCT standards, health providers should be trained to recognize high-quality tests, informed about the standards, and continuously updated about new products that meet those standards. Client flipcharts could include the photos and brand names for tests that meet these standards and could motivate the client to ask to see that specific test. This could help to motivate both POCT implementers and clients to demand quality and transparency and for the Peruvian government (DIGEMID, the oversight body for drugs and medical instruments, and the Ministry of Health) to provide the high-quality tests.

Finally, study participants – who are also future consumers of new POCTs for maternal health – affirmed the importance of thinking beyond traditional models of POCT implementation. To date, discussion about ANC-related POCTs has focused on their provision at health establishments [13,14]. For example, health establishments were the primary implementation point for syphilis POCTs in ANC in several countries including Peru [7,15]. However, at one site in this multi-country study, community health workers (CHWs) successfully implemented syphilis POCTs with remote indigenous populations in Brazil where there were no health facilities or laboratories and no previous syphilis screening [15]. Additionally, a recent policy-focused review of POCTs for infectious diseases in low- and middle-income countries proposed the creation of target product profiles for five different settings – homes, communities, primary care facilities, peripheral laboratories and hospitals. These target profiles vary by setting and include a spectrum of technologies, from simple to more sophisticated, and a range of POCT administrators, from lay people to highly trained professionals [16]. Participants in this study affirmed the importance of continuing to think beyond traditional models and considering the implementation of POCTs for ANC outside of the health system and by individuals who are not health workers. Participants emphasized that key characteristics of POCT administrators are people with good rapport with community members who, with training and monitoring/quality control, can implement POCTs. They mentioned that even community members themselves could administer POCTs with the appropriate supports. This model could function well given that participants mentioned that a significant proportion of pregnant women have some ability to pay and that they and their partners would be willing to pay for POCTs implemented closer to their homes, with shorter waiting times at all steps in the testing process, and with other key factors such as good treatment, good information and good linkage to follow-up. This non-traditional model, which could be implemented close to or at pregnant women’s homes, would not be a replacement for the health system. Instead, it could lessen the burden on an already-overburdened health system and link pregnant women to the health system, together with the results of key ANC diagnostic tests that could go directly into their medical records.

Finally, local partners at different levels could promote this non-traditional, community-based model. In our study, pregnant women’s male partners proved to be champions of maternal health and they are also directly involved in decision making around diagnostic tests for their partners, particularly if the tests have a cost. Male community members could be part of efforts to promote POCTs. Another important local partner may be the private sector. Female participants in all sites had visited the private sector for pregnancy-related diagnostic testing and particularly, for ultrasounds. If a community-based model is sought out, it would be essential to ensure that the private sector is offering high-quality tests and the tests that women require for health reasons versus – for example – for institutional profit reasons. Similarly, efforts and policies would need to be in place to designate that test results from the private sector should be recognized in the public and semi-public sectors, to avoid duplication of efforts and resources.

## Conclusions

The diversity of perspectives provided by our study participants, who are also future consumers of POCTs for ANC, have made it possible to begin developing innovative social business models for the non-traditional implementation of POCTs that will improve the health and well-being of pregnant women in rural and urban low-resource settings across the diverse regions of Peru. We expect that data collected during the pilot implementation of these models will provide important evidence for scale-up implementation in Peru and in other low-resource settings worldwide.

## Competing interests

The authors declare that they have no competing interests.

## Authors’ contributions

AMB and PJG conceived of the study and participated in its design. AMB, LN and MZ developed the data collection instruments and performed the data analysis. AMB drafted the manuscript. All authors read and approved the final manuscript.
